# The Application of Brain Organoids: From Neuronal Development to Neurological Diseases

**DOI:** 10.3389/fcell.2020.579659

**Published:** 2020-10-22

**Authors:** Yikai Shou, Feng Liang, Shunliang Xu, Xuekun Li

**Affiliations:** ^1^The Children’s Hospital, School of Medicine, Zhejiang University, Hangzhou, China; ^2^The Institute of Translational Medicine, School of Medicine, Zhejiang University, Hangzhou, China; ^3^National Clinical Research Center for Child Health, Hangzhou, China; ^4^Department of Neurosurgery, Second Affiliated Hospital, School of Medicine, Zhejiang University, Hangzhou, China; ^5^Department of Neurology, The Second Hospital, Cheeloo College of Medicine, Shandong University, Jinan, China

**Keywords:** brain organoids, neuronal development, neurological disease, 3-D, stem cell

## Abstract

Brain organoids are derived from induced pluripotent stem cells and embryonic stem cells under three-dimensional culture condition. The generation of an organoid requires the self-assembly of stem cells, progenitor cells, and multiple types of differentiated cells. Organoids display structures that resemble defined brain regions and simulate specific changes of neurological disorders; thus, organoids have become an excellent model for investigating brain development and neurological diseases. In the present review, we have summarized recent advances of the methods of culturing brain organoids and the applications of brain organoids in investigating neurodevelopmental and neurodegenerative diseases.

## Introduction

The current knowledge of the human brain is mostly based on post-mortem corpse brain specimens, mainly due to ethical issues. Animal models, including non-human primates, have several discrepancies compared to the human brain. These deficiencies have posed great challenges for studying the development of the human central nervous system (CNS) and related diseases (Adams et al., 2019). The advent and the rapid progress of stem cell technology, including human embryonic stem cells (hESCs) and human induced pluripotent stem cells (hiPSCs), have provided new insights of human brain development and neurological diseases (Thomson et al., 1998; Takahashi and Yamanaka, 2006; Takahashi et al., 2007).

On the basis of stem cell technology, the emergence of three-dimensional (3-D) organoids has attracted great attention in regenerative medicine. Brain organoid is a type of organoid that reproduces specific brain structures and has been used to simulate different human brain regions, including the midbrain (Jo et al., 2016; Monzel et al., 2017), hippocampus (Sakaguchi et al., 2015), pituitary gland (Ozone et al., 2016), hypothalamus (Qian et al., 2016), and cerebellum (Muguruma et al., 2015). Thus, brain organoids become an excellent model for investigating brain development and the mechanisms of related diseases (Lancaster and Knoblich, 2014b; Kelava and Lancaster, 2016; Kretzschmar and Clevers, 2016; Di Lullo and Kriegstein, 2017; Benito-Kwiecinski and Lancaster, 2019). Very recently, with the advances of gene editing, single cell sequencing, and other cutting-edge technologies, new vitality has been injected into the field and has brought unprecedented possibilities for modeling neurological diseases *in vitro*.

In this review, we first summarized the new advances in culture techniques and generation protocols of brain organoids. We then highlighted the applications of brain organoids in investigating human brain development, neurological diseases, and cerebral toxicity exposure.

## Methodological Progress in the Culture of Brain Organoids

To generate brain organoids, embryoid bodies (EBs) derived from human pluripotent stem cells (hPSCs) are generally embedded into an extracellular matrix (such as Matrigel) and then cultivated in a rotating bioreactor to promote tissue amplification and neural differentiation (Kadoshima et al., 2013; Qian et al., 2016). Some studies have also generated human cortical spheroids and organoids from pluripotent stem cells using a 3D culture system without embedding into extracellular matrices, and the neurons produced also display functional maturity and synaptogenesis (Pasca et al., 2015; Xiang et al., 2017). During culture, small molecules and growth factors are usually supplemented and promote hPSCs to form specific structures of the different brain regions (Qian et al., 2019). As the starting cell population, neural progenitors (Xu R. et al., 2019) and neuroepithelial stem cells (Monzel et al., 2017) are also used to generate organoids.

### Prolonged Culture Time

**FIGURE 1:**
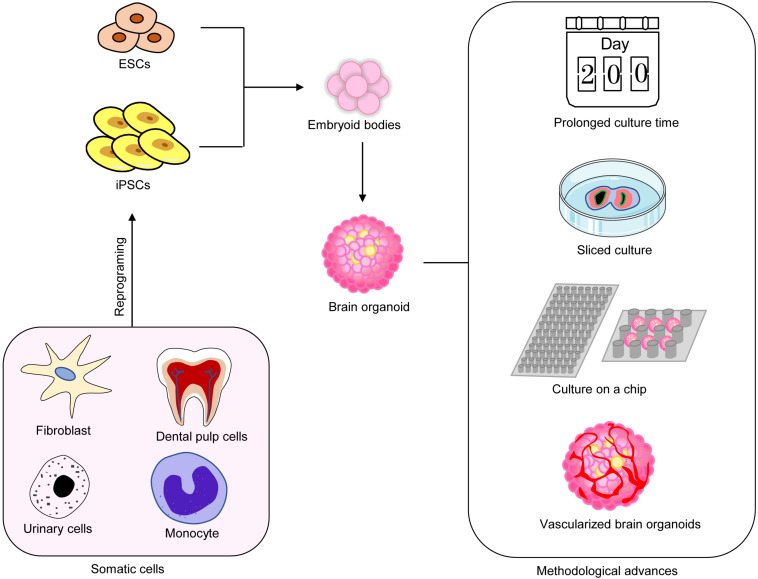
Recent methodological advances in brain organoids. Multiple methods have been used to improve the maturation of brain organoids.

Short time-cultured brain organoids mainly contain astrocytes, neurons, and neural stem/progenitor cells but usually lack mature oligodendrocytes and functional mature neurons. With longer culturing time, calcium activity can be detected after culturing for 50 days, and more cells display calcium activity (Pasca et al., 2015; Qian et al., 2016; Li et al., 2017). Spontaneous excitatory post-synaptic currents can also be detected in organoids cultured for 4 months (Li et al., 2017). The expression of the markers for mature astrocytes and neurons, synapses, and dendritic spines can be observed from organoids cultured for 6 months or longer (Quadrato et al., 2017). Long-term culturing not only promotes the maturation of neurons but also enhances the growth and differentiation of glial cells. It has been reported that brain organoids cultured for 229 days *in vitro* are filled with abundant glial cells positive for GFAP and GLT1 (Renner et al., 2017). Thus, long-term cultivation promotes the maturation of brain organoids and better captures the development of the human brain (Figure 1).

### Sliced Brain Organoid Culture

Organotypic slice culturing has greatly improved the oxygen supply of organoid tissues and reduced the formation of hypoxic cores. In 2019, Lancaster’s group has adopted air–liquid interface culture techniques, which improve the survival rate of neurons and the growth of axons and promotes the formation of circuits and the output of functional neurons (Giandomenico et al., 2019). More recently, it has been found that sliced neocortical organoid system can promote the continuous neurogenesis and dilation of the cortical plate; the cortical plate has distinct upper and deep cortical layers, which captures the neocortex in late human pregnancy and eliminates the restriction of growth and diffusion of brain organoids to some extent (Qian et al., 2020). These results indicate that brain organoids sliced culture can be used to study human-specific advanced cortical development and disease-related mechanisms (Figure 1).

### Culture on Microfluidic Chips

Microfluidic and engineering techniques have made great contributions to improving the repeatability and the uniformity of brain organoid cultures. The specificity performance of these technologies is that they can simplify the course of organoid cultures and provide better geometric constraints and environmental control (Ao et al., 2020). Microfluidic chips simplify the manufacturing process of brain organoids, and micro-pillar array devices have been used for *in situ* formation of plentiful brain organoids (Zhu et al., 2017). Brain organoids on-a-chip system exhibits definite neuronal differentiation, regionalization, and cortical tissue, which summarize the key features of early development of the human brain (Wang et al., 2018). This system has been applied to mimic brain wrinkling and to explore the effects of physical forces on the development of organoids (Karzbrun et al., 2018). Recently, a novel microfluidic platform with several unique advantages has been established (Ao et al., 2020). The device has combined *in situ* air–liquid interface culture to establish an integrated workflow and to support a one-stop assembly and culture platform for brain organoids (Ao et al., 2020). With the continuous advances and improvement of bio-engineering technology, brain organoid cultures can become a low-cost, short-time, and mass-culturable technology.

### Vascularized Brain Organoids

Brain organoids generated by traditional methods usually lack microvasculature, which is considered to be detrimental to organoids. Under long-term culture conditions, the absence of the vascular system restricts oxygen and nutrient transporting to the innermost parts of brain organoids, therefore inducing apoptosis and cell death in the inner zones (Lancaster and Knoblich, 2014a; Yin et al., 2016; Heide et al., 2018). Furthermore, the lack of functional vasculature affects the differentiation and maturation of neuronal/glial progenitor cells (Shen et al., 2004). Vascularized human cortical organoids (vhCOs) are generated through ectopic expressing human ETS variant 2 (ETV2). Moreover, 20% of cells infected with ETV2 in hCO is optimal to form vhCOs. On day 30 of culture, CD31^+^ endothelial tubes appear, and a more complex network of CD31^+^ vessel structure is observed on day 70 (Shen et al., 2004). In addition, vhCO also has more obvious blood–brain barrier characteristics, manifested by the unique expression of tight junction markers (such as α-ZOI), astrocyte and pericyte proteins, and transporters (Cakir et al., 2019).

Very recently, another co-culture system of hPSCs and human umbilical vein endothelial cells has been used to generate vascularized organoids, which display a well-developed tubular vascular structure (Shi et al., 2020). Vascularized organoids show reduced apoptosis and hypoxia of cells and more synaptic connections and establish vascular connections after transplantation *in vivo* (Cakir et al., 2019; Shi et al., 2020).

### Specialized Brain Organoids

With the advances of technologies, more types of cells, including oligodendrocytes (OLs) and interneurons, have been used to generate organoids. OLs are essential for brain development, including myelinating and electrically insulating neuronal axons for impulse propagation, as well as to provide nutrition and metabolic support to neurons. However, single-cell sequencing results indicate that regular cortical organoids lack oligodendrocyte progenitor cells (Quadrato and Arlotta, 2017; Sloan et al., 2017).

To overcome these issues, Madhavan et al. (2018) have exposed developed organoids to oligodendrocyte growth factors to induce oligodendrocyte progenitors and myelinating OLs in cortical spheroids. Promyelinating drugs can promote oligodendrocyte production and myelination and recapitulate the phenotypes of myelination defect diseases (Madhavan et al., 2018). Kim et al. (2019b) have applied the OLIG2-green fluorescent protein (GFP) stem cell reporter line to generate forebrain organoids, and the production of OLs can be monitored by GFP signal. With their protocol, the maturation of OLs is accelerated and can be observed as early as 9 weeks after organoid formation (Kim et al., 2019b). Paşca’s group has developed another protocol to culture organoids, which produce OLs, astrocytes, and neurons (Marton et al., 2019). Their protocol applies a set of small molecules and growth factors and can be used to study the development of OLs, myelination, and the interaction with other major cell types in the central nervous system (Marton et al., 2019).

Interneurons play a key role in regulating the activity of cortical networks. Xiang et al. (2017) have generated organoids to recapitulate the development of human medial ganglionic eminence (MGE). These organoids contain functional cortical interneurons, neuronal networks, and key ventral brain domains, which are similar with the developing MGE and cortex (Xiang et al., 2017).

## Applications of Brain Organoids as Disease Models

**FIGURE 2:**
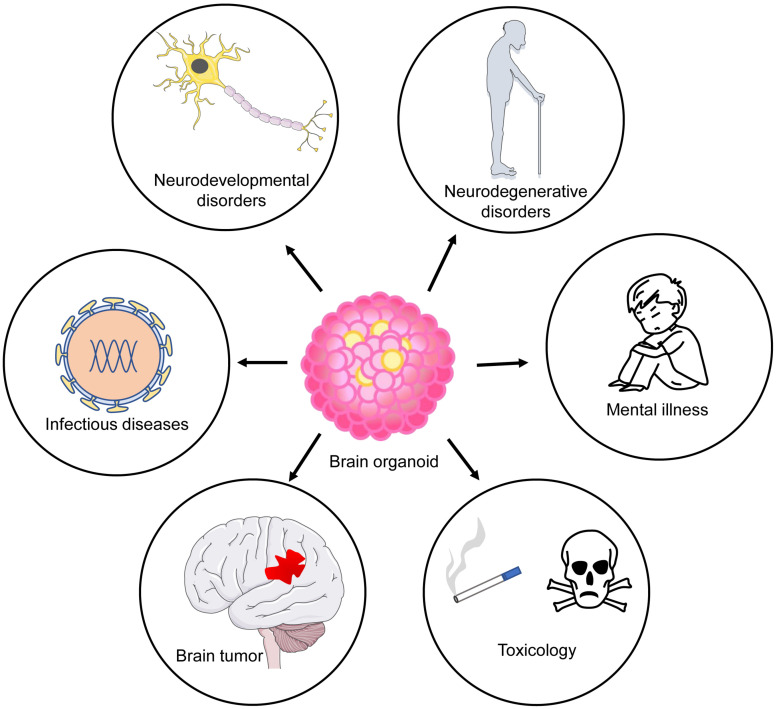
Application of brain organoids as disease models. Brain organoids have been used to model neurodevelopmental and degenerative diseases.

Previous studies have shown that brain organoids can recapitulate some key features of the human brain, including cellular distribution and organization, physiological structure, electrical activities, and neuronal networks (Lancaster et al., 2013; Pasca et al., 2015; Qian et al., 2016). Therefore, brain organoids have become a unique model to explore the mechanisms of neurological disorders (Figure 2).

### Neurodevelopmental Disorders

#### Primary Microcephaly

Primary microcephaly, also known as true microcephaly or autosomal recessive primary microcephaly, is mainly caused by genes that regulate the assembly of centrosomes and cilium caused by autosomal recessive mutations including *MCPH1*, *ASPM*, *WDR62*, *CDK5RAP2*, *CPAP*, and *CENPJ*. Currently, specific congenital microcephaly brain organoids carrying mutations of *CDK5RAP2*, *CPAP*, *ASPM*, and *WDR62* have been established, respectively (Lancaster et al., 2013; Li et al., 2017; Quadrato and Arlotta, 2017; Zhang et al., 2019).

Lancaster et al. (2013) have established a cerebral organoid model of primary microcephaly. A patient’s somatic cells with heterozygous truncation mutations of *CDK5RAP2* have been reprogrammed to iPSCs. After having been transferred to neural induction, the neuroepithelial tissue generated from the patient iPSCs is smaller than that of the control group. The generated cerebral organoids contain fewer radial glial stem cells (RGs) and more neurons, suggesting that the loss of *CDK5RAP2* leads to premature neural differentiation at the expense of progenitor cells (Lancaster et al., 2013). Centrosomal-P4.1-associated protein (CPAP protein) is related to microcephaly, and its mutation can cause Seckel syndrome and microcephaly. Brain organoids derived from a Seckel syndrome patient with *CPAP* mutation display a smaller size and premature neuronal differentiation (Gabriel et al., 2016). Furthermore, Seckel organoids show increased number and length of cilium compared to those of the control organoids, suggesting a delayed breakdown of cilium (Gabriel et al., 2016). These findings reflect the role of cilium in the maintenance of neural progenitor cells (NPCs) and indicate that *CPAP* is a negative regulator of cilium length. *WDR62* mutant iPSCs-generated organoids show delayed cilia decomposition, lengthening and cell cycle progression, reduced proliferation, and premature differentiation of NPCs (Zhang et al., 2019). The mechanism study shows that *WDR62* interacts with *CEP170* and promotes *CEP170* to locate in the matrix of primary cilia, where *CEP170* recruits the microtubule depolymerization factor KIF2A to decompose cilium (Zhang et al., 2019). These findings provide new insights into the pathogenesis of primary microcephaly.

*ASPM* mutant microcephaly organoids display less neuroepithelial tissues, fewer ventricular radial glial cells and outer radial glial cells (oRGs), and poor lamination (Li et al., 2017). Reduced maturation and electrical activity are observed in the *ASPM* mutant organoids, which is related to congenital mental retardation in patients with *ASPM* mutations. Wang L. et al. (2020) have conducted related verifications with whole-exome sequencing and uncovered microcephaly-related mutations of *NARS1* in more than 5,000 people with neurodevelopmental disorders. They have generated cortical brain organoids with *NARS1* mutations and found that patient-derived organoids display a smaller size, decreased proliferation, and cell cycle defects of RGs (Wang L. et al., 2020).

#### Acquired Microcephaly

In addition to the primary microcephaly caused by chromosomal mutations, external environment, infection, and other factors can also cause secondary microcephaly. The most studied is microcephaly caused by the infection of Zika virus (ZIKV). ZIKV particles can bind to cell membranes, localize in mitochondria and cellular vesicles, and lead to cell death and inhibit the formation of neurospheres (Garcez et al., 2016). Qian et al. (2016) have developed a forebrain organoid and modeled ZIKV exposure at different stages of pregnancy. The infection of ZIKV at the early stage of organoids (day 14) significantly decreases the thickness and the size of the VZ zone, while the size of the lumen of the ventricular structure significantly increased (Qian et al., 2016), which are very similar with the clinical phenotypes of central ventricular dilatation in fetus brain infected with ZIKV (Driggers et al., 2016).

#### Lissencephaly

Miller Dieker syndrome (MDS) is the most serious form of classical lissencephaly, which is characterized by reduced brain size, craniofacial deformities, mental retardation, and seizures. Brain organoids derived from MDS patients show increased apoptosis and reduced vertical divisions (Bershteyn et al., 2017; Iefremova et al., 2017). The defects of radial migration of neurons, cell autonomy, and delayed oRG cell-specific cytokinesis are also observed (Bershteyn et al., 2017; Iefremova et al., 2017). These mitotic defects of oRG may be involved in the pathogenesis of human lissencephaly. The forebrain organoids derived from MDS patients also display a shift from symmetrical to asymmetrical cell division of ventricular radial glial cells (vRGCs) (Iefremova et al., 2017). Furthermore, they have also observed severe changes in the organization of the ventricular niche in MDS organoids, including the low compactness of vRGC tissues and the disorderly positioning of cells retracted from the apical membrane (Iefremova et al., 2017). These phenotypes can be rescued by regulating the N-cadherin/β-catenin pathway, suggesting an important function of Wnt signaling in MDS.

#### Autism Spectrum Disorders

Autism spectrum disorder (ASD) is a neurodevelopmental disorder and induced by diverse pathogenic factors, such as genetic mutation, epigenetic modifications, and environmental factors. Cortical organoids derived from ASD patients display preferred differentiation toward GABAergic neurons, but no changes of glutamatergic neurons, resulting in the imbalance of glutamate/GABA neuron, which is resulting from the altered expression of FOXG1 (Mariani et al., 2015). A multiomics study shows that iPSC-derived cortical organoids show a similar transcriptome and epigenome pattern with isogeneic fetal brain tissue, especially between 5 and 16 post-conceptional weeks (Amiri et al., 2018). This study has also revealed 49,640 active enhancers important for cortical neuron specification (Amiri et al., 2018), and differentially expressed genes are highly related with the Wnt/β-catenin signaling pathway (Wang et al., 2017). *CHD8* is an ASD-related gene, and cerebral organoids derived from iPSCs with *CHD8* gene mutation show that *CHD8* regulates other ASD-related genes such as *TCF4* and *AUTS2*.

Macrocephaly/autism disorder represents a subset of ASD, and the loss of function of *RAB39B* mutation can cause macrocephaly, ASD, and epilepsy (Giannandrea et al., 2010). *RAB39B* mutant cerebral organoids have a larger volume than the normal control and display impaired differentiation and excessive proliferation of NPCs. Mechanistically, *RAB39B* deletion induces the over-activation of PI3K-AKT-mTOR signaling, and the inhibition of PI3K-AKT-mTOR signaling can rescue the phenotypes (Giannandrea et al., 2010).

#### Periventricular Heterotopia

The development of the neocortex in mammals is a highly coordinated process that depends on the precise generation, migration, and maturation of neurons. Periventricular heterotopia is one of the most common forms of cortical developmental malformations and is closely related to *DCHS1* and *FAT4* (Cardoso et al., 2009). The somatic cells of patients carrying mutations of *DCHS1* or *FAT4* were used to construct iPSCs and brain organoids. The morphology of the processes of NPCs appears to be neatly arranged and straight in normal organoids. However, neuronal processes are often destroyed and exhibit a distorted morphology in *FAT4*-mutant or KO organoids (Klaus et al., 2019).

#### Neonatal Hypoxic Injury

Neonatal hypoxic injury (NHI) is the most common reason for neonatal death and disability. Survivors usually suffer with cerebral palsy, epilepsy, and cognitive impairment (Mwaniki et al., 2012). Brain organoids of NHI have been established and used to examine the effects of different oxygen concentrations. The results show that hypoxia inhibits the expression of genetic markers (e.g., *FOXG1*, *DCX1*, *CLIP2*) for forebrain, OLs, glial cells, and the migrating cortical neurons, which could be alleviated by minocycline. Furthermore, minocycline also restrained apoptosis induced by hypoxia in brain organoids (Boisvert et al., 2019).

#### Down Syndrome

Down syndrome (DS) is the most common genetic cause of learning difficulties and is the most common form of dementia in people under 50 years old. Factors causing DS dementia are mainly divided into two major types: neurodevelopmental and neurodegenerative factors.

As a common neurodevelopmental disorder, the imbalance of excitatory and inhibitory neurotransmission predominantly contributes to the cognitive deficits of DS. DS organoids produce abundant OLIG2^+^ NPCs and a variety of CR^+^ and SST^+^ GABAergic neurons (Xu R. et al., 2019). Of note is the fact that there are some discrepancies between 2D and 3D cultures: OLIG2^+^ NPCs can generate different subtypes of neuron in 3D culture, while only CR+ neurons can be obtained in 2D culture (Xu R. et al., 2019). These findings suggest that OLIG2 is a potential target for DS in the clinic.

DS patients also display some phenotypes observed in AD patients. Gonzalez et al. (2018) have found that organoids derived from DS patients and familial AD (fAD) patients can spontaneously exhibit amyloid plaque deposition and Tau hyperphosphorylation, which are more significant than fAD. Furthermore, around 30% of DS patients have delayed onset of dementia, and the triplication of *BACE2* may be the underlying mechanism (Wiseman et al., 2015). In line with these findings, the trisomic level of *BACE2* protects T21-hiPSC organoids from early AD-like amyloid plaque pathology. Their results suggest the physiological role of *BACE2* as a suppressor for AD, and *BACE2* can serve as a therapeutic target (Alic et al., 2020).

### Neurodegenerative Disorders

#### Alzheimer’s Disease

Alzheimer’s disease (AD) is the most common neurodegenerative disease and is characterized by cognitive decline, behavioral impairment, and progressive deterioration of physical functions. Choi et al. (2014) have established a 3D culture system with human neural stem cell *via* overexpressing *APP* and *PSEN1* and successfully observed the aggregation of amyloid beta and tau pathology, suggesting the advantage of 3D culture (Kim et al., 2015). Continuous and spontaneous Aβ aggregation is observed in human neural organoids derived from fAD patients. At the later stage of culturing, fAD organoids show a significantly high immunoreactivity of pTau compared to the control group. β- and γ-secretase inhibitors reduce the pathologic changes induced by amyloid β and Tau phosphorylation in fAD organoids (Raja et al., 2016). Therefore, brain organoids could be a versatile tool for screening therapeutic compounds for neurodegenerative diseases.

Another recent study shows that 3D brain-like tissues infected with herpes virus can directly produce a new model of AD, which can simulate the formation of amyloid plaques, gliosis, neuroinflammation, and impaired functionality in the pathological process of AD (Cairns et al., 2020).

#### Parkinson’s Disease

Parkinson’s disease (PD) is the second most common neurodegenerative disease after Alzheimer’s disease and is characterized by the loss of dopaminergic neurons in the substantia nigra, of which typical motor symptoms include bradykinesia, muscle stiffness, resting tremor, and postural and gait disorders. The current cellular and animal models of PD have some limitations to mimic the phenotypes of PD. For example, animals with genetic mutations including *LRRK2* mutations cannot clearly show evidence of progressive midbrain dopamine neuron loss or Lewy body formation (Chesselet et al., 2008; Kim et al., 2019a).

Human midbrain-specific organoids derived from sporadic PD patients with *LRRK2*-G2019S mutation contain midbrain dopaminergic neurons (mDAN), but the number and the complexity of mDAN in *LRRK2* organoids are decreased than those of the control group, which is consistent with the phenotype of PD patients (Kordower et al., 2013). Kim et al. (2019a) have introduced the heterozygous *LRRK2-G2019S* point mutation into hiPSC using CRISPR-Cas9 technology and generated the isogeneic midbrain organoids (MOs). They found that, in the mutant MO, the neurite length of dopaminergic neurons was shortened, and the expression of corresponding markers including *TH*, *AADC*, *VMAT2*, and *DAT* was also reduced (Kim et al., 2019a). Moreover, other PD-related pathological signatures such as increased aggregation and abnormal clearance of α-synuclein are also found in MOs. The gene expression profiling data show that the mutant MOs have many similarities with that of a PD patient’s brain tissue. They find that *TXNIP* is specifically upregulated in mutant Mos, and the inhibition of *TXNIP* can suppress the phenotype induced by LRRK2 in MOs, so *TXNIP* may be related to LRRK2-related sporadic PD patients (Kim et al., 2019a). All these findings provide important insights into the pathophysiology of PD development.

In addition to sporadic PD, MOs derived from idiopathic PD patients show an altered expression of LIM homeobox transcription factor alpha (early) and tyrosine hydroxylase (late) markers (Chlebanowska et al., 2020). Several key genes relating to idiopathic forms of PD, such as neuronal marker genes *TH*, *PTX3*, *LMX1A*, and *FOXA2*, have been identified (Chlebanowska et al., 2020). Recently, Kwak et al. (2020) have developed a new type of midbrain-like organoids, which have stable and homogeneous structures and can produce mDANs, as well as other neuronal subtypes and glial cells. These results suggest that MOs can serve as an excellent model for both sporadic and familiar PD.

### Brain Tumor

Glioblastoma (GBM) is the most malignant form of glioma, accounting for 54% of all gliomas (da Hora et al., 2019). Cerebral organoids have been used to model primary human GBM *in vitro*. Organoids were co-cultured with glioma stem cells (GSCs) to obtain a cerebral organoid glioma (GLICO) model. Organoids co-cultured with glioma stem cells show that GSCs metastasize to the inner zones of organoids and deeply infiltrated and proliferated in host tissues, forming tumors closely related to patients with GBM (Linkous et al., 2019), suggesting that the GLICO model reflects well the malignant characteristics of GBM.

Medulloblastoma (MB), which occurs predominantly in the cerebellum, is one of the most common and aggressive malignant brain tumors in children and induce a high rate of mortality (Rutkowski et al., 2010). Group 3 MB is one of the most aggressive MB subgroups, which is characterized by *c-MYC* up-regulation. The results from 3 MB cerebellar organoid show that *OTX2/c-MYC* is a new driving gene required for 3 MB tumorigenesis. The treatment of EZH2 inhibitor tazemetostat can inhibit *OTX2/c-MYC* tumorigenesis in organoids (Ballabio et al., 2020). Therefore, human cerebellar organoids can be effectively used to explore the roles of genetic mechanisms in glioma patients.

### Infectious Diseases of the CNS

#### Neurotropic Virus Infections

As mentioned above, ZIKV is a neurotropic virus that preferentially infects human NPCs. The development of brain organoids has greatly promoted the study of neurotropic viruses and provided an alternative method for animal and 2D cell culture models of ZIKV infection (Antonucci and Gehrke, 2019). One study shows that enoxacin exposure can prevent ZIKV infection and avoid the microcephalic phenotype in brain organoids. This study also discovered the physiological importance of RNAi-mediated antiviral immunity in the early stages of human brain development, revealing new strategies to enhance RNAi’s resistance to human congenital viral infection (Xu Y. P. et al., 2019).

In addition to screening drugs for the prevention and treatment of ZIKV infection, the neurotoxicity of ZIKV has been used to explore its potential efficacy and mechanism as an oncolytic virus to GBM. The findings show that ZIKV preferentially targets GSCs in GBM cortical organoids, showing effective antitumor effects over time. In preclinical studies, the application of GBM organoids enhances selective tumor targeting and may provide positive implications for oncolytic virus therapies (Zhu et al., 2020).

#### Cerebral Malaria

Malaria is a parasitic disease caused by *Plasmodium*. Cerebral malaria is one of the clinical manifestations of malaria and usually accompanied by severe neurological complications (Nanfack et al., 2017). Malaria causes hemolysis and produces a by-product called heme, which promotes the apoptosis and spontaneous differentiation of iPSCs and induces the changes of brain injury-related biomarkers, such as the increased expression of CXCL-10, CXCR3, and BDNF and the decreased expression of ERBB4 in organoids. They find that neuregulin-1 had neuroprotective effects on heme-treated organoids (Harbuzariu et al., 2019). Thus, this brain organoid model can be used to study the effects of hemolysis (not limited to malaria infection) on fetal brain development.

### Mental Illness

#### Schizophrenia

Schizophrenia is one of the most intractable diseases in brain health, with complex genetic/environmental causes, molecular neuropathology, and neurodevelopmental origins. Due to the functional and structural differences of brain regions in rodents and human being, it is challenging to observe the phenotypes of mental illness in rodents (Wang M. et al., 2020).

Forebrain organoids derived from schizophrenia patients with *DISC1* mutations show the altered proliferation of radial glial cells (Ye et al., 2017). The interaction between *DISC1* and *NDEL1* plays an important role in maintaining the neural stem cell population during the development of the human forebrain (Ye et al., 2017). Cerebral organoids with an isogenic *DISC1* mutation show the over-activation of the WNT signaling pathway (Srikanth et al., 2018). Morphological analysis shows that *DISC1* organoids show a chaotic structural morphology and impaired proliferation, which can be rescued by WNT antagonism (Srikanth et al., 2018). Brain organoids derived from schizophrenia iPSCs show decreased proliferation and neuronal development and reduced expression of FGFR1 protein in cortical cells, accompanied by the loss of nFGFR1 signaling (Stachowiak et al., 2017). Blocking and depleting FGFR1 with the antagonist PD173074 in the control organoids can cause cortical growth arrest similar to schizophrenia. In turn, it also shows that rebuilding FGFR1 in developing cortical neurons can inhibit developmental abnormalities (Stachowiak et al., 2017).

## Toxin Exposure of the CNS

In addition to modeling neuronal development and neurological disorders, brain organoids can be used to evaluate the effects of acute and chronic toxin exposure.

### Prenatal Exposure

#### Prenatal Nicotine Exposure

Previous studies have shown that nicotine exposure during pregnancy may be associated with neurodevelopmental impairment and behavioral disorders in children. Wang et al. (2018) have used a brain organoid-on-a-chip system to simulate the nervous system exposed to prenatal nicotine. Their findings show that nicotine exposure can cause premature differentiation and apoptosis of neurons in brain organoids, also inhibiting neurite outgrowth and the structural development of the cortex, which is manifested as the decreased expression of forebrain markers (*PAX6* and *FOXG1*). Their study indicates that brain organoids can be a useful model to study the effects of toxin on neuronal development.

#### Prenatal Methamphetamine Abuse

Methamphetamine (METH) is an addictive stimulant that causes temporary intense excitement. METH addicts may experience symptoms such as decreased hippocampal volume and memory loss (Chang et al., 2007; Du et al., 2015). To determine the effects of prenatal METH abuse on the human brain, 10-month-old brain organoids are exposed to METH for 1 week, followed by scRNA-seq analysis. The results show that METH can significantly alter the expression of neuroinflammatory and cytokine-related genes and affect the proliferation, differentiation, and cell death of NSCs (Dang et al., 2020).

#### Prenatal Cannabis Exposure

In addition to METH, the effects of prenatal cannabis exposure on brain development were studied with human brain organoids. They demonstrated that prolonged exposure to tetrahydrocannabinol could alter the neonatal brain VZ/SVZ ratio, downregulate the cannabinoid receptor type 1 receptors, and inhibit neurite outgrowth and spontaneous neuronal activity (Ao et al., 2020).

## Future Challenges of Brain Organoids

It is a breaking advance to culture human “brain” in a lab dish and visualize it daily. Brain organoids *bona fide* provide an excellent model for us to understand the development, aging, and evolvement of the human brain, and dramatic progress has been made in brain organoids during the past decade. Up to today, brain organoids have been used in exploring mechanisms for neurological diseases, drug screening, etc.

Although brain organoids display a significant advantage relative to conventional 2D culture, researchers do realize a few issues in the field. First, to generate and culture brain organoids is technically challenging and requires multiple reagents. It will be even more challenging to harvest healthy organoids if cultured for a longer time. Second, there are some variations between organoids even from the same chamber. This variation will definitely affect the results that compare the size and the volume between the control and patient-derived organoids. Therefore, it is necessary to improve the culture methods and increase the reproducibility. Third, the dynamic cellular composition, structure, maturity, crosstalk between types of cells, etc., occur during brain development and aging. It is still a great challenge to mimic well the complexity of the human brain with organoids in a spatiotemporal pattern. Brain organoids for some brain structures such as the hippocampus and the cerebellum have not been generated yet. Furthermore, organoids generated for neurodegenerative diseases including AD only very partially simulate the pathological features of AD. To resolve these issues, we would expect technical advances.

## Author Contributions

YS, FL, SX, and XL wrote the manuscript. All authors contributed to the article and approved the submitted version.

## Conflict of Interest

The authors declare that the research was conducted in the absence of any commercial or financial relationships that could be construed as a potential conflict of interest.
